# Utilization of Recycled Brick Aggregate in Cementitious Composites: Mechanical Performance, Cement Matrix Solidification Effect and Life Cycle Assessment

**DOI:** 10.3390/ma19183847

**Published:** 2026-09-10

**Authors:** Vojtěch Václavík, Jana Seidlerová, Rudolf Hela, Tomáš Dvorský, Klára Křížová, Tomáš Široký, Miroslav Škopán, Jaroslav Kašpárek, Jiří Frühbauer, Lubomír Keim

**Affiliations:** 1 Department of Environmental Engineering, Faculty of Mining and Geology, VSB—Technical University of Ostrava, 17. Listopadu 15/2172, 708 00 Ostrava, Czech Republic; tomas.dvorsky@vsb.cz; 2 Nanotechnology Centre, VSB—Technical University of Ostrava, 17. Listopadu 15/2172, 708 00 Ostrava, Czech Republic; jana.seidlerova@vsb.cz; 3 Institute of Technology of Building Materials and Components, Faculty of Civil Engineering, Brno University of Technology, Veveří 331/95, 602 00 Brno, Czech Republic; rudolf.hela@vut.cz (R.H.); klara.krizova@vut.cz (K.K.); 4 Department of Mining Engineering and Safety, Faculty of Mining and Geology, VSB—Technical University of Ostrava, 17. Listopadu 15/2172, 708 00 Ostrava, Czech Republic; tomas.siroky@vsb.cz; 5 Institute of Automotive Engineering, Faculty of Mechanical Engineering, Brno University of Technology, 616 69 Brno, Czech Republic; skopan@fme.vutbr.cz (M.Š.); kasparek@fme.vutbr.cz (J.K.); jiri.fruhbauer@vutbr.cz (J.F.); 6 Research Institute for Building Construction—Certification Company, Ltd., Prazska 810/16, 102 00 Prague 10, Czech Republic; l.keim@vups.cz

**Keywords:** recycled brick material, recycled aggregate, cement composites, solidification, environmental assessment, life cycle assessment

## Abstract

The growing production of construction and demolition waste has been stimulating the need to look for sustainable alternatives to natural aggregate used in the production of cement composites. This study evaluates the potential use of recycled brick material as a substitute for natural coarse aggregate in terms of environmental safety, mechanical properties, the solidification effect of the cement matrix, and life cycle assessment (LCA). Recycled brick materials sourced from four recycling yards were characterized using physical, chemical, and leaching tests. Cement composites containing 50%, 70%, 80%, and 100% replacement of natural coarse aggregate with recycled brick material were subsequently designed. The results demonstrated significant variability in the environmental properties of the recycled materials depending on their origin. Compressive strength after 28 days ranged from 31.8 to 36.2 MPa, and all mixtures met the requirements for strength classes C20/25 to C25/30 according to ČSN EN 206+A2. At the same time, a significant solidification effect of the cement matrix was confirmed, leading to a reduction in the concentrations of dissolved substances by 68.0–80.1% and sulfates by 97.8–99.2% compared to the original recycled material. A life-cycle assessment demonstrated a gradual reduction in the environmental impacts as the proportion of recycled brick material increased, with the best results achieved by a mixture with 100% replacement of natural aggregate. The results confirm that recycled brick material can be safely used as a full-fledged substitute for natural coarse aggregate while maintaining the required mechanical properties and reducing environmental impacts.

## 1. Introduction

Construction and demolition waste represents one of the most significant material flows generated in the European Union. At the same time, there is a growing demand for an efficient use of natural resources, a reduction in waste generation, and the implementation of circular economy principles in the construction industry. One promising option for the material reuse of construction and demolition waste is its processing into recycled aggregate that can be used in the production of cement composites [1,2,3,4].

Recycled brick material generated during the demolition of masonry structures represents a significant group of these materials. Compared to natural aggregate, it is characterized by higher porosity, higher water absorption, and lower bulk density, which significantly affects the properties of both fresh and hardened concrete mixtures [1,2,3,4]. Debieb and Kenai [1] demonstrated that the use of both coarse and fine recycled brick material leads to changes in the physical properties of concrete and an increase in the required mixing water content. Cachim [2] found that the mechanical properties of concrete are significantly influenced by the extent to which natural aggregate is replaced by recycled brick material, with an increasing proportion of recycled brick material generally leading to a reduction in bulk density and compressive strength.

Despite its higher porosity and water absorption, a number of studies have shown that recycled brick material can be successfully used in the production of structural concrete. González-Fonteboa et al. [3] verified the feasibility of using recycled brick material in precast prestressed concrete elements and demonstrated that, with proper design of the concrete mixture, parameters meeting the requirements of construction practice can be achieved. Uddin et al. [4] further demonstrated that a significant factor influencing the properties of concrete is the particle size of the recycled brick aggregate, which has a direct impact on both the strength characteristics and the long-term behavior of the composite.

In recent years, research has focused not only on the use of recycled aggregate but also on the fine fractions produced during the crushing of brick materials. Liu et al. [5] demonstrated that recycled brick powder exhibits partial pozzolanic activity and can be used as a supplementary cementitious material. According to Hu et al. [6], the use of brick powder can contribute to improving the microstructure of the cement matrix, increasing the efficiency of secondary raw material utilization, and simultaneously reducing Portland cement consumption.

Current research is increasingly focused on studying the microstructure of cement composites containing recycled materials. Particular attention is paid to the transition zone between the recycled aggregate grains and the cement matrix (Interfacial Transition Zone—ITZ), which significantly influences the mechanical properties and durability of the composites. He et al. [7] demonstrated that appropriate surface treatment of recycled aggregate can improve the bond between the recycled aggregate grains and the cement matrix, thereby increasing the strength and durability of the composite.

In addition to mechanical properties, the durability of concrete containing recycled brick material is also an important parameter. Zong et al. [8] found that the use of recycled brick material affects the permeability of the cement matrix and the transport properties of concrete. Ge et al. [9] further demonstrated the effect of fine recycle brick material on the shrinkage and long-term durability of self-compacting concretes. Casapino-Espinoza et al. [10] and Zhang et al. [11] also addressed the issue of the physical and mechanical properties of concrete containing recycled brick material, confirming the possibility of using these materials in cement composites while maintaining the required technical parameters.

In addition to the technical properties, it is also essential to evaluate the environmental aspects of using recycled materials. Wong et al. [12] note that the use of recycled bricks helps reduce the consumption of natural resources, limit aggregate mining, and decrease the amount of construction waste deposited in landfills. Consequently, increasing attention is currently being paid to the comprehensive assessment of the environmental impacts of construction materials through life cycle assessment (LCA) methods, which make it possible to quantify the benefits of using recycled materials in terms of raw material consumption, energy intensity, and greenhouse gas emissions.

At the same time, it is essential to assess the potential release of selected substances from recycled materials into the environment. Butera et al. [13] and Galvín et al. [14] demonstrated that construction and demolition waste may contain components that could potentially affect the quality of the aquatic environment, with their mobility depending on the material’s composition, the pH of the environment, and the leaching conditions.

One specific area is the ability of the cement matrix to limit the mobility of potentially hazardous substances through stabilization and solidification processes. Cement hydration products are capable of immobilizing selected elements and compounds through two principal mechanisms: physical encapsulation within the hardened cement matrix and chemical binding or incorporation into hydration products. These processes reduce the mobility and leachability of potentially hazardous substances. Chen et al. [15] described these mechanisms of cement stabilization and solidification and demonstrated the importance of cement-based systems for the immobilization of potentially hazardous substances.

Although the use of recycled brick material in cement composites has been studied extensively in recent years, most published works focus primarily on the physical and mechanical properties, microstructure, or durability of concrete. Significantly less attention has been paid to the simultaneous evaluation of technical parameters, environmental safety, and the ability of the cement matrix to immobilize potentially mobile components contained in the recycled material. At the same time, only a limited number of studies combine the evaluation of physical and mechanical properties with environmental assessment using leaching tests and life-cycle assessment methods.

The aim of this work is therefore to comprehensively assess the potential for using recycled bricks as a substitute for natural aggregate in cement composites. The focus is on evaluating the physical and mechanical properties of experimental concretes, the environmental characteristics of the input recycled material, assessing the solidification effect of the cement matrix through leaching tests, and evaluating the environmental benefits of using recycle brick material applying the life cycle assessment (LCA) method.

## 2. Materials and Methods

### 2.1. Recycled Bricks

**Figure 1:**
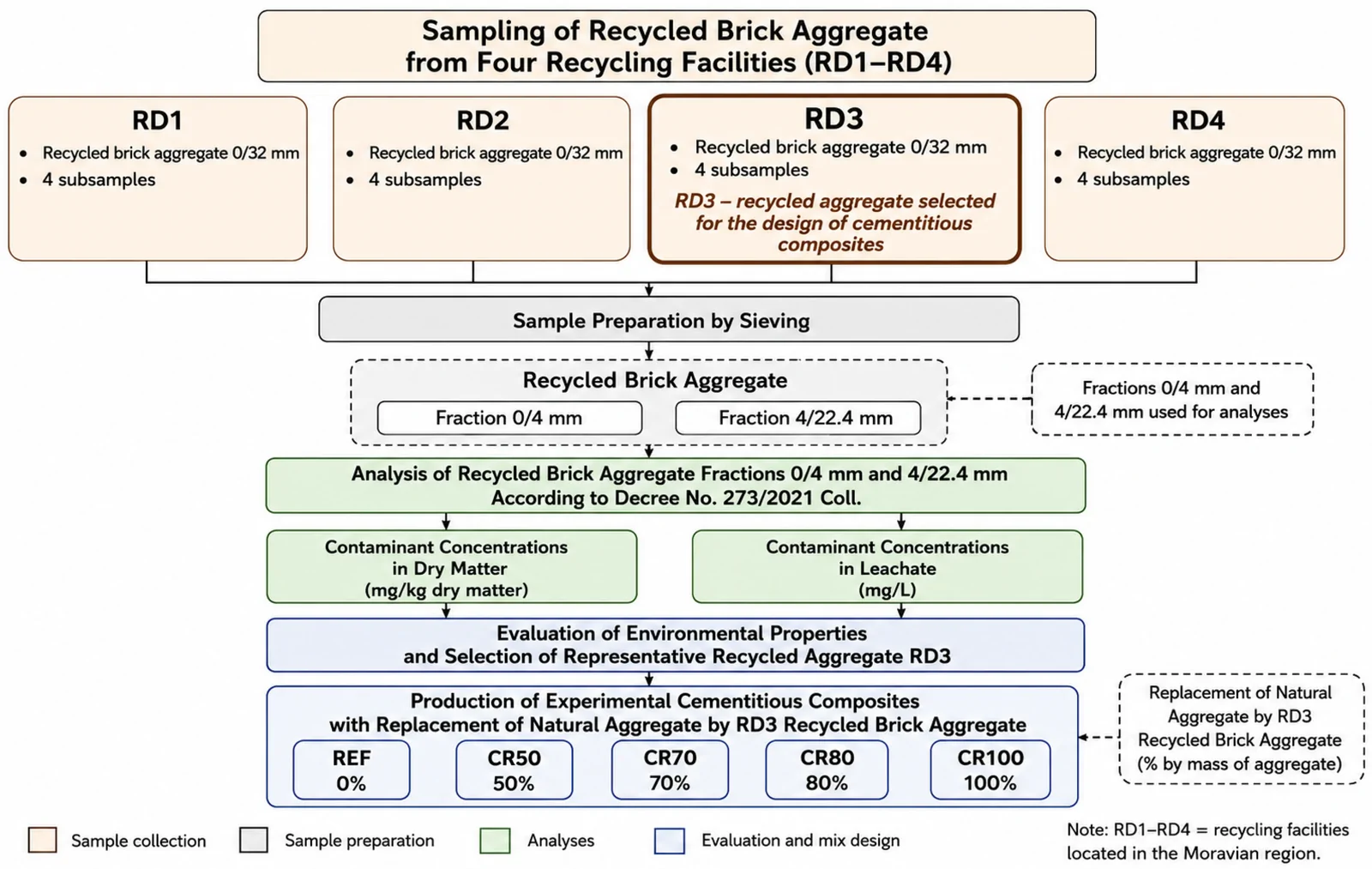
Experimental workflow for sampling, environmental assessment, selection of representative recycled brick aggregate (RD3), and design of cementitious composites (CR—recycled brick aggregate).

The recycled brick material used in the experimental part of the research came from a recycling yard designated RD3. The selection of this material was preceded by a study of the properties of recycled brick materials from four recycling yards in Moravia (RD1–RD4). The procedure for sampling, preparation, and evaluation of the samples is illustrated in Figure 1.

Representative samples of recycled brick aggregate with a 0/32 mm fraction were collected from each recycling yard. The collected samples were subsequently processed in the laboratory through a sorting process into the 0/4 mm, 4/22.4 mm, and 22.4/63 mm fractions. The 0/4 mm and 4/22.4 mm fractions were used for the environmental assessment.

For all samples, the concentrations of pollutants in the dry matter and in the aqueous extract were determined in accordance with the requirements of Decree No. 273/2021 Coll. The aim of the assessment was to evaluate the environmental suitability of individual sources of recycled brick material for their subsequent use in cement composites.

Based on the results of the survey, recycled brick material from the RD3 recycling yard was selected for the experimental production of cement composites. The selection of RD3 was based on the results of the environmental assessment of recycled brick materials from all four recycling yards (RD1–RD4). RD3 was deliberately selected because it exhibited elevated concentrations of selected environmental parameters in both the dry matter and the aqueous extract, particularly EOX, dissolved substances, and sulfates. Thus, RD3 represented a suitable material for evaluating not only its potential use as a substitute for natural aggregate but also the ability of the cement matrix to reduce the mobility of potentially problematic components through solidification/stabilization. This material was subsequently used as a substitute for natural coarse aggregate in the production of the experimental mixtures. In the designed mixtures, natural aggregate was replaced by recycled brick material in amounts of 50%, 70%, 80%, and 100% by weight of the aggregate, while the reference mixture was produced exclusively with natural aggregate.

Recycled brick aggregate with a 0/22 mm particle size, sourced from the RD3 recycling yard (see Figure 1), was used to produce the experimental cement composites. The basic physical properties of the recycled aggregate were determined in accordance with European standards for testing aggregates. The bulk density and water absorption were determined according to EN 1097-6 [16]. The granulometric composition was determined according to EN 933-1 [17]. The shape index was determined according to ČSN EN 933-4 [18].

The determined bulk density of the recycled brick material was 2120 kg·m^−3^, and the water absorption (WA_(24)_) reached 10.6%. Compared to natural aggregate, the recycled brick material exhibits a lower bulk density and significantly higher water absorption, which is a result of the porous structure of fired ceramic materials.

**Figure 2:**
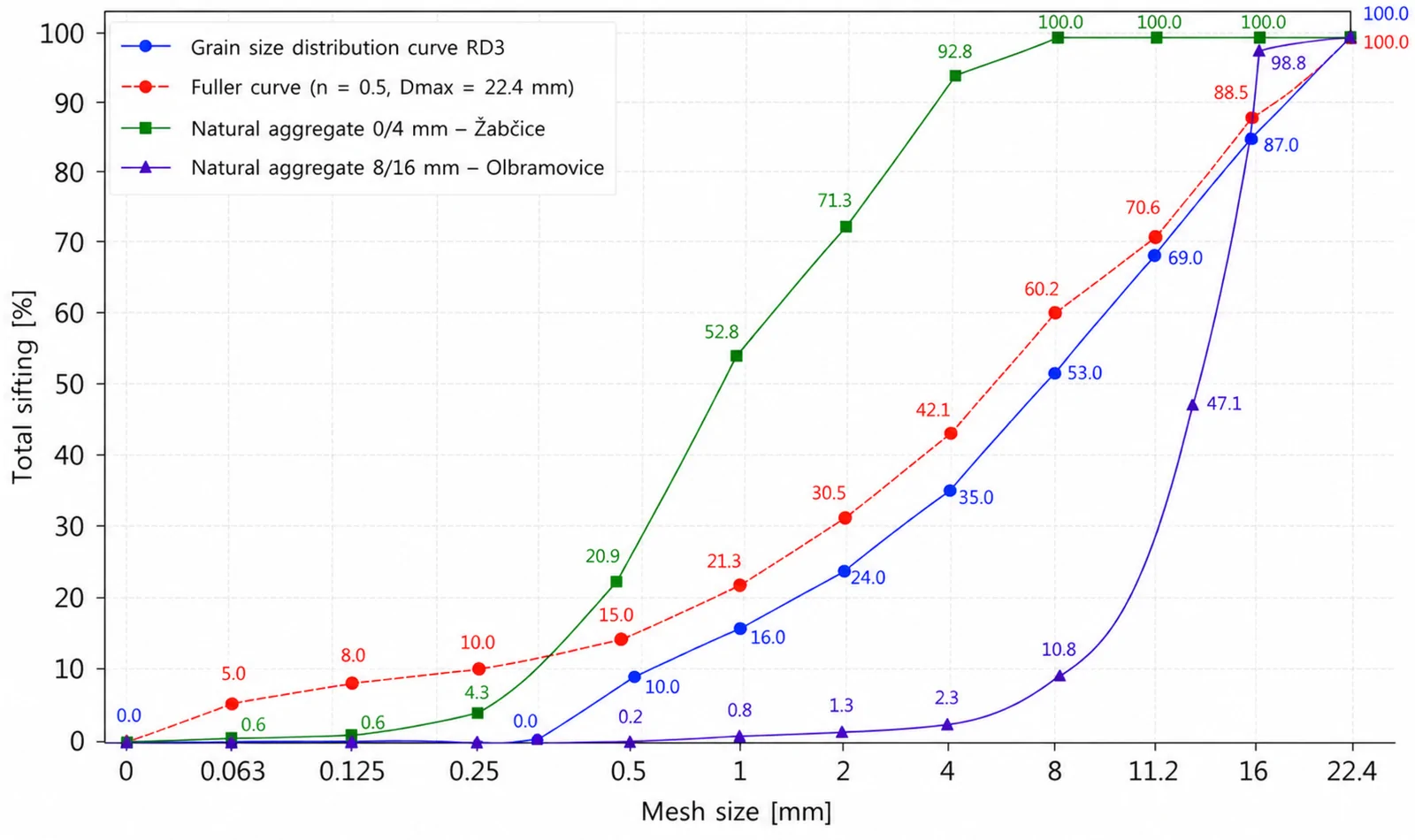
Grain size distribution curve for RD3 recycled brick aggregate, 0/22.4 mm fraction (blue curve), compared with the Fuller curve (red curve).

The granulometric composition of the recycled material is shown in Figure 2. For comparison, the graph also includes the Fuller curve, which represents the ideal granulometric composition of aggregate. The results of the sieve analysis show that the recycled rick material exhibits a continuous granulometric composition across the entire evaluated range of fractions, without any significant discontinuities or deficiencies in individual size fractions. The content of fine particles smaller than 0.25 mm was approximately 5%, while the proportion of particles smaller than 4 mm reached 35%. The coarser fractions were found to be the most abundant, with 53% of the material passing through an 8-mm sieve and 87% passing through a 16-mm sieve. When compared to the Fuller curve, the particle size distribution curve of the recycled brick material shows slight deviations, which are characteristic of recycled aggregates produced by crushing construction and demolition waste. The increased proportion of coarser fractions can be attributed to the nature of the crushed ceramic material. Nevertheless, it can be concluded that the granulometric composition of the recycled material is favorable for its use in cement composites, as it allows for good workability of the mixture while simultaneously reducing the need for additional adjustments to the aggregate’s granulometric composition.

Figure 2 also presents the particle-size distributions of the commercially supplied natural aggregates used in the mixture design, namely the 0/4 mm fraction from Žabčice and the 8/16 mm fraction from Olbramovice. The 0/4 mm natural aggregate is characterized by a continuous distribution within the fine particle-size range, with 92.8% passing the 4 mm sieve and 71.3% passing the 2 mm sieve. In contrast, the 8/16 mm natural aggregate represents the coarse fraction, with 98.8% passing the 16 mm sieve, 47.1% passing the 11.2 mm sieve, and 10.8% passing the 8 mm sieve. The inclusion of these grading curves in Figure 2 provides a direct comparison between the particle-size distribution of the natural aggregates and the recycled brick aggregate RD3 used in the experimental mixtures.

The shape index was determined for the 4/8 mm, 8/16 mm, and 16/22 mm fractions. In all evaluated fractions, very similar shape index values were found in the range of 12.1–12.5%, which corresponds to category SI15. According to EN 933-4, the SI15 category indicates that the shape index of the aggregate does not exceed 15%. The results confirm the favorable grain shape of the recycled material in terms of its use in cement composites. The results confirm the favorable grain shape of the recycled material in terms of its use in cement composites.

A summary of the basic physical properties of the RD3 recycled rick aggregate used in the experimental part of the research is presented in Table 1.

### 2.2. Natural Aggregate

Quarry-mined aggregate with a 0/4 mm fraction from the Žabčice sand pit (PÍSEK ŽABČICE spol. s r.o., Czech Republic) was used as fine natural aggregate in the production of the experimental cement composites. The aggregate met the requirements of the EN 12620+A1 standard [19]. The aggregate for the preparation of the concrete was supplied with a valid declaration of performance.

According to the manufacturer’s declared properties, the aggregate had a grain size category of GF85, a bulk density of 2.56 Mg·m^−3^, and a water absorption rate of WA24 at 1.19%. The fine particle content corresponded to category f3. At the same time, a very low chloride content (0.0001%) and a sulfate content corresponding to category AS0.2 were declared. The aggregate also met the requirements for humic particle content and total sulfur. A summary of the basic properties of the natural aggregate used is presented in Table 2.

### 2.3. Cement

Portland cement CEM I 42.5 R (sc), manufactured by Českomoravský cement, a.s., Mokrá plant, Mokrá-Horákov, Czech Republic., was used to produce the experimental cement composites with a solidification effect based on recycled brick material. The cement met the requirements of the EN 197-1 standard [20] and is characterized by a rapid increase in strength, high initial strength, and high ultimate strength, making it suitable for the production of concrete in both standard and higher strength classes. The selection of CEM I 42.5 R was primarily based on the objective of evaluating the solidification effect of the cement matrix on potentially leachable components originating from recycled brick aggregate. Its high clinker content and well-defined composition provide a consistent cementitious matrix suitable for assessing the immobilization of these components. In addition, its rapid strength development and high final strength provide a suitable basis for evaluating the mechanical performance of mixtures with different recycled brick aggregate replacement levels. The basic physical, mechanical, and chemical properties of the cement used, as declared by the manufacturer, are listed in Table 3.

### 2.4. Plasticizing Admixture

The CHRYSO® Quad 920 (Chryso Polska Sp. z o.o., Błonie, Poland) superplasticizing admixture, based on polycarboxylate ethers (PCE), was used in the production of the experimental cement composites. The admixture was selected in consideration of the use of recycled brick material, which, compared to natural aggregate, is characterized by higher water absorption and a larger specific surface area, which can negatively affect the workability of fresh concrete mixtures.

The CHRYSO Quad series of admixtures is designed primarily for concretes containing recycled aggregate or aggregate with a high content of fine particles. Their use allows for a reduction in the amount of mixing water required while maintaining the desired consistency of the mixture, improving the rheological properties of fresh concrete, and achieving the desired mechanical properties of hardened concrete. The admixture was added to the mixing water in the amount specified during the design of the individual experimental mixtures.

The use of the superplasticizing admixture contributed to achieving the required workability of concretes containing different proportions of recycled brick aggregate while limiting the amount of additional mixing water required.

### 2.5. Mixing Water

Drinking water from the public water supply system was used to produce the experimental cement composites with a solidifying effect based on recycled brick material.

### 2.6. Design of the Experimental Formulas

Experimental cement composites were designed in such a way to assess the effect of replacing natural aggregate with recycled brick material on the physical and mechanical properties of concrete, and to evaluate the solidification effect of the cement matrix on potentially hazardous substances contained in the recycled material. The composition of the individual experimental mixtures is shown in Table 4.

For comparison, a reference C20/25 mixture containing exclusively natural aggregate was included in the experimental programme. The aggregate skeleton of the reference mixture consisted of natural fine aggregate 0/4 mm and natural coarse aggregate 8/16 mm. In the mixtures containing recycled brick aggregate, the natural coarse aggregate fraction 8/16 mm was completely replaced by recycled brick aggregate RD3, while the natural fine aggregate fraction 0/4 mm was progressively replaced as the recycled brick aggregate content increased. In contrast, the mixtures containing recycled brick aggregate were intentionally designed using recycled brick aggregate with a continuous 0/22 mm grading. This approach was selected on the basis of the preceding experimental programme, which showed that the inclusion of the fine fraction of recycled brick material contributed to achieving the required workability, reduced the consumption of natural fine aggregate, and enabled more complete utilization of the recycled brick material. Therefore, the experimental mixtures were not designed as a simple one-to-one replacement of an individual natural coarse aggregate fraction by an equivalent recycled coarse fraction, but as a progressive replacement of the natural aggregate skeleton by recycled brick aggregate with a continuous 0/22 mm grading.

In addition to the reference mixture, four experimental mixtures containing recycled brick aggregate were designed, with recycled brick aggregate contents of 50%, 70%, 80%, and 100% by weight of the aggregate. The cement content was maintained at 305 kg·m^−3^ in all recycled-aggregate mixtures, whereas the amount of mixing water varied according to the proportion of recycled brick aggregate and the required workability. The cement content was maintained at 305 kg·m^−3^ in all mixtures, whereas the amount of mixing water varied according to the proportion of recycled brick aggregate and the required workability. Consequently, the nominal water-to-cement ratios (w/c) were 0.87, 0.81, 0.80, and 0.89 for mixtures containing 50%, 70%, 80%, and 100% recycled brick aggregate, respectively. The cement content was maintained at 305 kg·m^−3^ in all recycled-aggregate mixtures, whereas the amount of mixing water varied according to the proportion of recycled brick aggregate and the required workability. The different water contents were necessary primarily due to the porous structure and higher water absorption of recycled brick aggregate compared with natural aggregate, which directly affected the water demand of the individual mixtures. Consequently, the nominal water-to-cement ratios (w/c) were 0.87, 0.81, 0.80, and 0.89 for mixtures containing 50%, 70%, 80%, and 100% recycled brick aggregate, respectively. The designed mixture formulas made it possible to assess the effect of increasing recycled brick content on the properties of fresh and hardened concrete and, at the same time, to verify the ability of the cement matrix to stabilize and immobilize the substances of interest present in the recycled material.

### 2.7. Environmental Assessment of Recycled Brick Materials

The environmental properties of recycled brick materials were assessed in accordance with the requirements of Decree No. 273/2021 Coll., on details regarding waste management [21]. The objective of the assessment was to evaluate the suitability of individual recycled materials for their subsequent use in construction materials and, at the same time, to obtain data for assessing the solidification effect of the cement matrix on potentially hazardous substances contained in the recycled material.

Samples of recycled brick materials collected from four recycling yards (RD1–RD4) were used for the evaluation; these samples underwent laboratory treatment and subsequent environmental analyses. For all samples, the concentrations of selected parameters were determined in the dry matter and in the aqueous extract. Concentrations in the dry matter were determined for the indicators listed in Table 5.1 of Decree No. 273/2021 Coll. [21], while the evaluation of aqueous extracts was conducted in accordance with the requirements of Table 5.2 of the same decree.

The analyses were focused primarily on determining the content of heavy metals, polycyclic aromatic hydrocarbons (PAHs), extractable organically bound halogens (EOX), dissolved substances, sulfates, fluorides, and other indicators relevant to the environmental safety of the recycled materials. The results obtained were used to compare the individual sources of recycled brick materials, select a representative RD3 recyclate for the experimental part of the research, and subsequently assess changes in environmental properties after incorporating the recyclate into a cement matrix.

### 2.8. Life Cycle Assessment (LCA)

An environmental assessment of the designed cement composites with varying shares of recycled brick material was conducted using the Life Cycle Assessment (LCA) method. The analysis was conducted in accordance with the requirements of ISO 14040 [22], ISO 14044 [23], EN 15804+A2 [24], and EN 16757 [25] for concrete and concrete products. The assessment was focused on comparing the environmental impacts of cement composites containing 50, 70, 80, and 100% recycled brick aggregate with a reference mixture using natural aggregate.

A volume of 1 m^3^ of cement composite was selected as the declared unit. The evaluation included the mixtures listed in Table 4, which differed only in the extent to which natural aggregate was replaced by recycled brick material, while maintaining a constant content of CEM I 42.5 R cement.

Specific data obtained from Environmental Product Declarations (EPDs) for CEM I 42.5 R cement and natural aggregate were used for the life cycle modeling, while data for recycled brick material, superplasticizing admixture, transportation, and energy inputs were taken from the Ecoinvent 3.11 database (https://ecoinvent.org/ecoinvent-v3-11/, accessed on 4 September 2026). The LCI was based on the quantities of the individual components specified for each concrete mixture and the corresponding processes from the Ecoinvent 3.11 database. Supplier-specific EPD data accounted for 77.5% of the total result, while database data represented 22.5%.

The assessment was conducted for the entire product life cycle, covering modules A1–A3 (material production and composite production), A4–A5 (transport and installation), B1–B7 (use phase), C1–C4 (end-of-life), and module D, which covers the potential benefits of material recycling after the structure’s end-of-life. To compare the designed mixtures, the results of the production phase (A1–A3)—which accounts for the dominant share of the total environmental impacts—were primarily evaluated.

In accordance with EN 15804+A2 [23], the main categories of environmental impacts were evaluated, including global warming potential (GWP), acidification, eutrophication, photochemical ozone formation, depletion of abiotic resources, consumption of fossil resources, and water use. The results of the assessment were subsequently used to evaluate the environmental benefits of replacing natural aggregate with recycled brick aggregate in cement composites with a solidification effect.

## 3. Results and Discussion

### 3.1. Environmental Properties of Recycled Brick Materials

An environmental assessment of recycled brick materials was conducted on samples collected from four recycling yards (RD1–RD4). Based on the results of analyses of substance concentrations in dry matter and in aqueous extracts, indicators were identified that most significantly influence the potential for using recycled brick material in construction materials. These included, primarily, the content of arsenic (As), chromium (Cr), extractable organically bound halogens (EOX), polycyclic aromatic hydrocarbons (PAHs), dissolved substances, sulfates, and fluorides.

The results of the evaluation of the concentrations of the monitored substances in the dry matter are presented in Section 3.1.1, while the results of the analyses of the aqueous extracts are presented in Section 3.1.2.

#### 3.1.1. Evaluation of Substance Concentrations in the Dry Matter of Recycled Brick Material

The results of the analyses of the concentrations of selected parameters in the dry matter of recycled brick materials collected from four recycling yards (RD1–RD4) are presented in Table 5. Only those parameters for which the limit values specified in Table 5.1 of Decree No. 273/2021 Coll. [21] were exceeded, or parameters showing significant differences between individual sources of recycled material are presented for the purpose of clarity. These included, in particular, arsenic (As), chromium (Cr), extractable organically bound halogens (EOX), and polycyclic aromatic hydrocarbons (PAHs), which represented the most significant environmental indicators in terms of the further use of recycled brick material in building materials.

The results presented in Table 5 show that the arsenic (As) concentrations in all evaluated recycled materials ranged from 9 to 16 mg·kg^−1^ of dry matter. The first limit value specified by Decree No. 273/2021 Coll. (10 mg·kg^−1^) was exceeded in most of the evaluated samples, while the second limit value (30 mg·kg^−1^) was not exceeded in any case. The highest concentration of arsenic was found in recycled material RD4 in the 0/4 mm fraction.

Significant differences between the individual sources of recycled material were observed in chromium (Cr) content. In all evaluated samples, chromium concentrations exceeded the first limit value of 100 mg·kg^−1^ of dry matter. At the same time, the second limit value of 200 mg·kg^−1^ was also exceeded in most samples, with the highest concentrations found in recycled material RD1, where the chromium content reached 477 mg·kg^−1^ in the 0/4 mm fraction and 333 mg·kg^−1^ in the 4/22.4 mm fraction. In most cases, higher chromium concentrations were found in the finer 0/4 mm fraction. The observed differences in chromium concentrations among the individual recycling yards confirm the heterogeneous nature of construction and demolition waste. A similar conclusion is reported by Butera et al. [13], who demonstrated that the chemical composition of recycled construction materials is significantly influenced by the origin of the input waste and the quality of its sorting.

Significant differences were found among the individual recycling yards with regard to extractable organically bound halogens (EOX). While no EOX were detected in the RD2 and RD4 recyclates, concentrations exceeding the first limit value of 1 mg·kg^−1^ were found in the RD1 and RD3 recyclates. The highest value was determined in the RD3 recyclate in the 4/22.4 mm fraction, where it reached 6 mg·kg^−1^ and also exceeded the second limit value of 2 mg·kg^−1^.

The greatest variability was observed for polycyclic aromatic hydrocarbons (PAHs). PAHs were not detected in most of the evaluated samples; however, concentrations of 9 mg·kg^−1^ were determined in the 0/4 mm fraction and 2 mg·kg^−1^ in the 4/22.4 mm fraction of recycled material RD4. The value found in the fine fraction of the RD4 recycled material exceeded both limit value I (3 mg·kg^−1^) and limit value II (6 mg·kg^−1^) as specified in Decree No. 273/2021 Coll.

Significant differences in EOX and PAH concentrations among the individual recycled materials confirm that organic contaminants can be significantly influenced by the composition of the input construction waste and the presence of undesirable impurities. Similar conclusions are also reported by Butera et al. [13], who point out the high variability in the content of organic substances in construction and demolition waste.

#### 3.1.2. Evaluation of Extracts from Recycled Brick Material

The results of the analyses of selected parameters in aqueous extracts from recycled brick materials collected from four recycling yards (RD1–RD4) are presented in Table 6. For clarity, only those parameters for which the limit values specified in Table 5.2 of Decree No. 273/2021 Coll. [21] were exceeded, or parameters showing significant differences between individual sources of recycled material, are presented. These included, in particular, dissolved substances, sulfates, and fluorides, which are key indicators in terms of the potential mobility of contaminants and the environmental safety of recycled construction materials.

The results presented in Table 6 show that the most significant exceedances of the limit values specified in Table 5.2 of Decree No. 273/2021 Coll. were found for dissolved substances (DS) and sulfates. The concentrations of dissolved substances in the 0/4 mm fraction ranged from 621 to 827 mg·L^−1^, thereby exceeding the limit value of 400 mg·L^−1^ in all evaluated recycled materials. In the coarser 4/22.4 mm fraction, the concentrations were lower, with the limit value exceeded only in recycled material RD3. The highest concentrations of dissolved substances were determined for recycled material RD3 in the 0/4 mm fraction (827 mg·L^−1^), while the lowest values were found for recycled material RD2.

A similar trend was also observed for sulfates. In all fine 0/4 mm fractions, sulfate concentrations significantly exceeding the limit value of 100 mg·L^−1^ were detected. The highest concentrations were found in recycled material RD3 (393 mg·L^−1^), followed by recycled materials RD1 and RD4. In the coarser 4/22.4 mm fractions, the limit values were exceeded in all evaluated samples as well, although the concentrations were significantly lower compared to the finer fractions. The results confirm that sulfates represent one of the key parameters in the environmental assessment of recycled brick materials.

The determined differences in the leachability of the monitored parameters confirm that the environmental assessment of the recycled construction materials cannot be based solely on determining the total content of substances in the dry matter. Galvín et al. [14] report that the mobility of contaminants is significantly influenced by the chemical conditions of the environment and the leaching test methodology used.

Fluoride concentrations in all evaluated samples were significantly lower than the limit value of 1.0 mg·L^−1^. The exception was recycled material RD1 in the 4/22.4 mm fraction, where a concentration of 1.07 mg·L^−1^ was determined, which slightly exceeded the limit value. In the other samples, fluoride concentrations were low or below the limit of quantification of the analytical method.

Based on the results of the leaching tests, it can be concluded that dissolved substances and sulfates were the decisive indicators for the environmental assessment of recycled brick materials. At the same time, it was confirmed that the finer 0/4 mm fraction showed higher mobility of the monitored substances than the 4/22.4 mm fraction in most cases, which is related to the higher specific surface area of fine-grained particles and more intense contact between the material and the leaching medium.

From the perspective of practical application, the higher leaching potential of the fine 0/4 mm fraction indicates that this fraction should be subjected to separate environmental assessment before further use. If the fine fraction meets the applicable environmental requirements, it can be directly utilized in suitable construction applications, for example as backfill material or for bedding and surrounding pipelines. If the environmental requirements are not met, direct use of the material should be avoided, and its incorporation into a cement matrix with a solidification/stabilization effect represents a suitable alternative. As demonstrated by the results of this study, the cement matrix can significantly reduce the mobility of selected soluble components contained in recycled brick material. Another potential utilization route is the use of suitably treated fine 0/4 mm recycled brick material as a supplementary component in concrete, which is also the subject of ongoing research by the authors. Therefore, separate environmental assessment of the fine fraction and selection of an appropriate utilization route according to its environmental properties should be considered an important part of quality control during recycled brick aggregate processing and can contribute to its safe and effective utilization in engineering practice.

### 3.2. Properties of Cement Composites with a Solidification Effect

The test results of the physical and mechanical properties of experimental cement composites with a solidification effect, in which natural aggregate was replaced with recycled brick material, are presented in Table 7. The properties of fresh concrete were evaluated, specifically its consistency, air content, and bulk density, as well as the bulk density of hardened concrete and compressive strength after 28 days of curing.

The consistency of the fresh mixtures ranged from 110 to 170 mm. The highest value was observed for the mixture with 70% RBA replacement (170 mm), whereas the mixtures with 50% and 80% replacement both reached 150 mm, and the mixture with 100% replacement exhibited the lowest value of 110 mm. The higher consistency observed for the 70% RBA mixture indicates that the relationship between the RBA replacement level and workability was not strictly monotonic. The workability of mixtures containing porous recycled brick aggregate is influenced by several interacting factors, particularly aggregate water absorption, the amount of mixing water, and aggregate packing. Although the nominal w/c ratios were 0.87, 0.81, 0.80, and 0.89 for the mixtures with 50%, 70%, 80%, and 100% RBA, respectively, these values do not directly represent the amount of water available for the rheological behaviour of the cement paste because part of the mixing water can be absorbed by the porous RBA. Therefore, the locally higher slump observed for the 70% RBA mixture is attributed to the combined effects of water availability, aggregate absorption, and aggregate packing rather than to the replacement level alone. The substantially lower slump observed at 100% replacement is consistent with the increased water demand associated with the higher amount of porous recycled brick aggregate.

The air content in fresh concrete ranged from 2.8% to 3.3%. As the proportion of recycled brick aggregate increased, a slight increase in the air content was observed, which can be attributed to the higher porosity of the recycled aggregate and the greater amount of fine particles present in the mixture.

The bulk density of fresh concrete ranged from 2030 to 2130 kg·m^−3^, with the highest values observed in the mixture with a 50% replacement of natural aggregate. A similar trend was also observed for the bulk density of hardened concrete, which ranged from 1950 to 2040 kg·m^−3^. The gradual decrease in bulk density is related to the lower bulk density of recycled brick material compared to natural aggregate.

The reference mixture containing only natural aggregate achieved a 28-day compressive strength of 34.5 MPa. The mixtures containing recycled brick aggregate achieved compressive strengths ranging from 31.8 to 36.2 MPa. The highest strength was observed in the mixture with 50% recycled brick aggregate (36.2 MPa), exceeding the reference mixture by approximately 5.8%, while the lowest value was found in the mixture with 70% recycled brick aggregate (31.8 MPa), approximately 7.0% below the reference value. The mixtures with 80% and 100% recycled brick aggregate reached 33.6 and 34.7 MPa, respectively, and thus remained close to the strength of the reference mixture. The non-monotonic development of compressive strength indicates that the mechanical response of the investigated composites cannot be explained solely by the recycled brick aggregate replacement level. The lowest compressive strength observed at 70% replacement (31.8 MPa) coincided with the highest measured consistency (170 mm), whereas the mixtures with 80% and 100% replacement exhibited lower consistency values and recovered compressive strengths of 33.6 and 34.7 MPa, respectively. This suggests that the observed strength variation resulted from an interaction between the water balance of the mixture, the high absorption capacity and internal porosity of the recycled brick aggregate, and the resulting structure of the hardened cement matrix. Although the nominal w/c ratios ranged from 0.80 to 0.89, these values do not directly represent the effective w/c ratio of the cement paste, because part of the mixing water can be absorbed into the porous brick particles. This water redistribution may affect local paste porosity, compaction, and hydration conditions and consequently contribute to differences in compressive strength. The measured air content increased only moderately from 2.8 to 3.3% with increasing recycled aggregate content and therefore does not by itself explain the strength minimum at 70% replacement. Similarly, the image analysis showed no disruption of the interfacial transition zone (ITZ) between the recycled brick aggregate and the cement paste and no visible cracking of the cement matrix in any of the investigated mixtures. Thus, the observed strength fluctuation cannot be attributed to an evident deterioration of the ITZ. Rather, it is considered to result from the combined effects of water redistribution, aggregate porosity, mixture workability, and the resulting pore structure of the cementitious composite. Since quantitative pore-structure analysis was not performed in this study, this interpretation should be considered mechanistic rather than a direct experimental verification of the individual contributions. Despite the observed fluctuations, all designed mixtures achieved compressive strengths exceeding 30 MPa. The results thus confirm that recycled brick material can be used as a substitute for natural aggregate in the production of cement composites without a significant negative impact on their mechanical properties. At the same time, it was experimentally verified that even with a high degree of substitution of natural coarse aggregate with recycled brick material, it is possible to design cement composites that meet the requirements of strength classes C 20/25 and C 25/30 according to EN 206+A2 [26].

To provide a broader context for the observed workability and compressive strength trends, the results of the present study were compared with selected previous investigations on concrete containing recycled brick aggregate. The comparison focuses on the RBA replacement level, water-to-cement ratio, workability, and 28-day compressive strength, as summarized in Table 8.

As summarized in Table 8, previous studies demonstrate that the mechanical response of concrete containing recycled brick aggregate is governed by several interacting parameters and cannot be interpreted solely on the basis of the RBA replacement level. In particular, Uddin et al. [4] reported a non-monotonic compressive-strength response depending on aggregate size and mixture-design parameters. This supports the interpretation that the strength minimum observed at 70% RBA replacement in the present study reflects the combined influence of mixture composition, water redistribution, aggregate characteristics, and workability rather than the replacement level alone.

The results obtained are consistent with the conclusions of a number of international studies examining the use of recycled brick material in cement composites. Debieb and Kenai [1] as well as Cachim [2] report that replacing natural aggregate with recycled brick material leads to a reduction in the bulk density of concrete and, at the same time, may cause a decrease in compressive strength due to the higher porosity and water absorption of the recycled aggregate. A similar trend was also observed in this study, where the bulk density of both fresh and hardened concrete decreased with an increasing proportion of recycled brick material, while the compressive strength remained above 30 MPa in all cases.

González et al. [3] and Uddin et al. [4] further report that, with suitable mixture design, mechanical properties meeting the requirements for structural concrete can be achieved even with a high proportion of recycled brick material. This fact was also confirmed in the experiment conducted, in which even a mixture with 100% replacement of natural aggregate achieved a compressive strength of 34.7 MPa. The results also support the conclusions of the review study by Wong et al. [12], according to which recycled brick material represents a promising alternative to natural aggregate, particularly with regard to reducing the consumption of primary natural resources and supporting the principles of the circular economy in the construction industry.

### 3.3. Solidification Effect of the Cement Matrix

To verify the ability of the cement matrix to immobilize potentially mobile components contained in recycled brick material, leaching tests were conducted on experimental cement composites containing 50%, 70%, 80%, and 100% replacement of natural aggregate with recycled brick material. The leaching tests were performed on hardened cement composites after 28 days of curing. Cylinders with a diameter of 40 mm and a height of 40 mm were used as the test specimens. The results were compared with the environmental properties of the reference recycled brick material RD3, which was selected for the design of the experimental mixtures based on the results presented in Section 3.1.1 and Section 3.1.2. An overview of the results is presented in Table 9.

The results presented in Table 8 show that the cement matrix significantly reduced the mobility of the substances under study in an aqueous environment. The most significant changes were observed for dissolved substances (DS) and sulfates, which had concentrations of 638 mg·L^−1^ and 318 mg·L^−1^, respectively, in the leachate from the initial recycled material. After incorporating the recycled material into the cement matrix, a significant decrease in the concentrations of these indicators was observed in all experimental mixtures. The concentrations of dissolved substances decreased to values ranging from 127 to 204 mg·L^−1^, thereby achieving a solidification efficiency in the range of 68.0–80.1%. At the same time, all experimental mixtures met the limit value of 400 mg·L^−1^ established by Decree No. 273/2021 Coll.

An even more pronounced effect was observed for sulfates. While the initial recycled material had a concentration of 318 mg·L^−1^, concentrations of only 2.57–6.88 mg·L^−1^ were determined for the cement composites. The solidification efficiency reached values of 97.8–99.2%, confirming the cement matrix’s very high ability to limit the mobility of soluble sulfate compounds. At the same time, all experimental mixtures met the limit value of 100 mg·L^−1^ by a significant margin.

For fluorides and total chromium, no exceedances of the limit values were detected even in the initial recycled material. After incorporating the recycled material into the cement matrix, the concentrations of these parameters were determined to be at or below the limit of quantification of the analytical method. The solidification efficiency of heavy metals was therefore not expressed quantitatively because their concentrations in the leachates of the cement composites were below the limits of quantification of the analytical methods used. For example, the concentration of total Cr decreased from 0.044 mg·L^−1^ in the original RD3 recycled brick material to <0.030 mg·L^−1^ in all cement composites. Therefore, an exact percentage reduction cannot be reliably calculated. Similarly, concentrations of arsenic, barium, cadmium, copper, mercury, molybdenum, nickel, lead, antimony, selenium, and zinc were also found to be below the limit of quantification or well below the limit values. Low values were also recorded for monohydric phenols and dissolved organic carbon (DOC), and none of the monitored parameters exceeded the statutory limits.

The results confirm the significant solidification effect of the cement matrix, which is capable of effectively limiting the mobility of soluble components and potentially hazardous substances contained in the recycled brick material. This phenomenon is generally attributed to the formation of cement hydration products, particularly C-S-H gels, ettringite, and other hydrates, which enable the physical encapsulation and, at the same time, chemical binding of the monitored substances. Similar conclusions are also reported by Chen et al. [15], who demonstrated a significant reduction in the mobility of inorganic contaminants due to their immobilization in the cement matrix.

Based on the results obtained, it can be concluded that the designed cement composites not only meet the mechanical property requirements specified in EN 206+A2 [26], but also offer significant environmental benefits by limiting the mobility of the substances under study. The cement matrix thus demonstrably exhibits a significant solidification effect and enables the safe use of recycled brick material in the production of cement composites.

### 3.4. Life Cycle Assessment

The results of the life cycle assessment of cement composites containing recycled brick material are presented in Table 10. To compare the designed mixtures, the results of production phases A1–A3—which account for the dominant share of the total environmental impacts of the assessed cement composites, were primarily evaluated. The subsequent life-cycle modules were assumed to be identical for all investigated mixtures and therefore do not depend on the recycled brick aggregate replacement level.

The results show that as the proportion of recycled brick material increases, there is a gradual reduction in most of the monitored environmental indicators. The most significant decrease was observed in fossil resources consumption, which fell from 1360 MJ in the mixture containing 50% recycled brick material to 1270 MJ in the mixture with a complete replacement of natural aggregate. At the same time, the global warming potential (GWP total) decreased from 299 to 293 kg CO_2_ eq., representing a decrease of approximately 2%. A similar trend was also observed for acidification, freshwater eutrophication, and water use.

To provide a more comprehensive evaluation of the environmental performance over the entire life cycle, Table 9 also presents the quantitative contributions of the subsequent life-cycle modules A4, A5, B, C1–C4, and D. Since identical scenarios were assumed for these stages for all investigated mixtures, their environmental impacts are independent of the recycled brick aggregate replacement level. The results confirm that the product stage (A1–A3) represents the dominant contribution to the environmental impacts, whereas the subsequent life-cycle modules generally show considerably lower contributions. Module D provides environmental credits resulting from the potential recovery and recycling of materials beyond the system boundary.

The relatively small differences between the individual mixtures are primarily due to the dominant influence of CEM I 42.5 R cement production on the overall environmental profile of the evaluated composites. The cement content was constant in all designed mixtures; therefore, the environmental benefit of replacing natural aggregate with recycled brick aggregate was only partially demonstrated. This conclusion is consistent with the results of a VÚPS study, according to which cement production is the decisive source of environmental impacts for cement composites, while the contribution of aggregate is significantly lower.

The other life cycle modules showed a significantly lower contribution to the overall environmental impacts compared to the production phase (A1–A3). Module A4 included the transport of input raw materials and other materials to the production site, and module A5 covered the production process of cement composites. Given the relatively short transport distances and the low energy intensity of the production operations, these modules accounted for only a limited share of the overall environmental profile of the evaluated mixtures.

Modules B1–B7 represent the use phase of a construction product, which includes operation, maintenance, repairs, renovation, possible replacement of the structure, and energy or water consumption over its service life. In the case of the evaluated cement composites, the impact of this phase was negligible, as no significant material or energy inputs were considered over the expected service life.

Modules C1–C4 covered the processes associated with the end of a structure’s service life, particularly demolition, transportation of the resulting waste, its treatment, and final disposal. Although the environmental impacts of this phase were lower than those of the production phase, they represent an important component of the overall life-cycle assessment of construction materials.

From the perspective of the circular economy, Module D was the most significant, as it reflects the potential environmental benefits resulting from the reuse of materials at the end of their life cycle. For the mixtures evaluated, this module represented an environmental credit associated with the possibility of replacing primary aggregate with recycled material. Module D thus partially offsets the environmental impact generated in previous life-cycle phases while simultaneously promoting the efficient use of secondary raw materials in the construction industry.

The results obtained are consistent with the conclusions of Wong et al. [12], who state that the use of recycled brick material leads to a reduction in the consumption of primary natural resources and an improvement in the environmental performance of concrete mixtures. Similarly, Hu et al. [6] note that the environmental benefits of recycled brick material are particularly evident in terms of conserving natural resources and reducing greenhouse gas emissions. At the same time, however, they point out that the overall environmental impact is significantly influenced by the recycling technology used, transportation distances, and the quality of the input data.

The results of this study confirm that the use of recycled brick material as a substitute for natural aggregate makes it possible to reduce the environmental impacts of cement composites while maintaining the required mechanical properties and the proven solidification effect of the cement matrix. From an environmental perspective, a mixture containing 100% recycled brick material appears to be the most advantageous, as it exhibited the lowest values for most of the evaluated environmental indicators.

### 3.5. Image Analysis

The goal of the image analysis was to identify the grains of natural aggregate and recycled brick material in the structure of cement composites and to assess the nature of the interfacial transition zone (ITZ) between the aggregate grains and the cement paste. The quality of the contact zone is one of the decisive factors influencing the mechanical properties and durability of concrete, as its disruption can lead to the formation and propagation of cracks.

**Figure 3:**
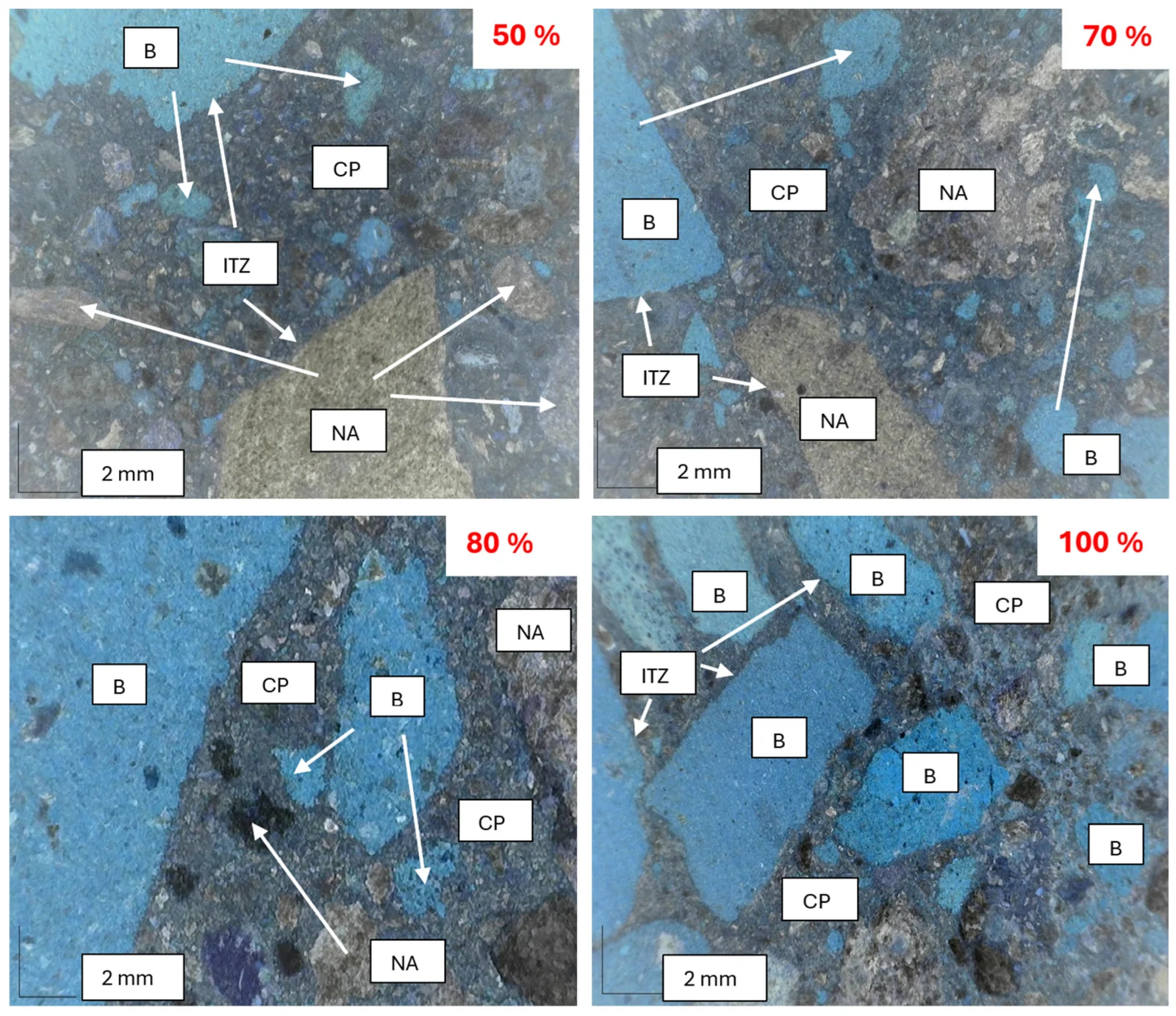
Imaging analysis of cement composites based on recycled brick material with a content of 50, 70, 80 and 100%; B—Brick, NA—Nature aggregate, ITZ—Interfacial Transition Zone, CP—Cement paste with aggregate.

Image analysis was performed on all cement composite mixtures in which natural aggregate was replaced with recycled brick material in the amount of 50, 70, 80, and 100% (Figure 3).

Based on the results of the image analysis, the following conclusions can be drawn:

- Grains of natural aggregate (NA) and grains of recycled brick material (B) can be clearly identified in the structure of the cement composites.
- In addition to hydration products, the cement paste (CP) also contains fine particles of natural aggregate and recycled brick material generated during their crushing.
- At the scale and resolution of the analyzed images, no visible disruption of the interfacial transition zone (ITZ) between the natural aggregate (NA) grains, recycled brick (B) grains, and cement paste (CP) was observed in any of the analyzed samples.
- At the scale and resolution of the analyzed images, no visible cracks in the cement paste, cracks or separation at the aggregate–cement paste interface, or visible structural discontinuities were observed.

### 3.6. Summary of the Main Research Findings

**Figure 4:**
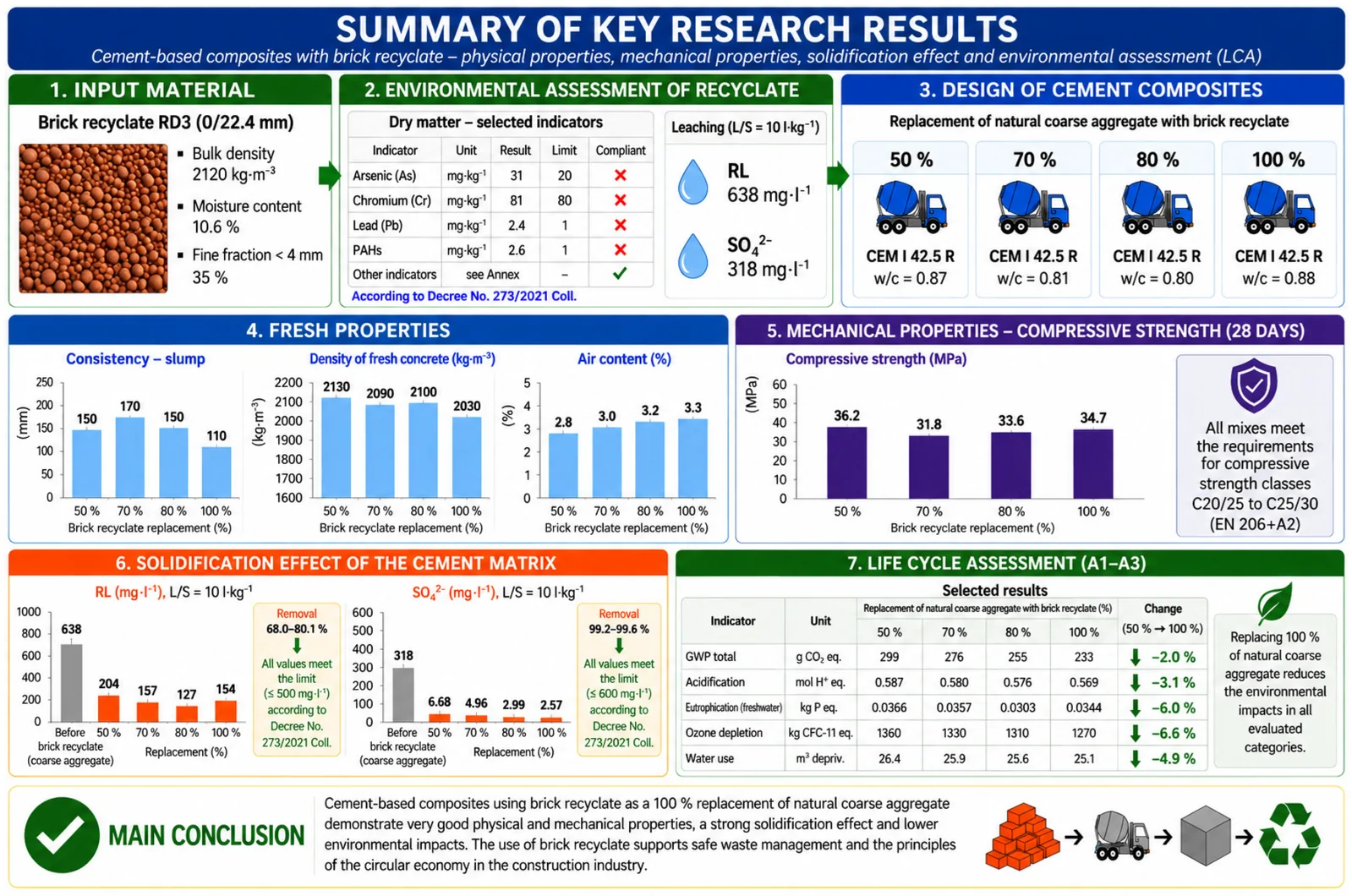
Summary of the main results of research focused on the use of recycled brick material as a substitute for natural coarse aggregate in cement composites.

For greater clarity and to highlight the interconnections among the results presented in Section 3.1, Section 3.2, Section 3.3 and Section 3.4, Figure 4 shows a summary diagram of the main research findings, including the environmental assessment of recycled brick material, the mechanical properties of cement composites, the solidification effect of the cement matrix, and the results of the life cycle analysis.

## 4. Conclusions

This study evaluated the potential use of recycled brick material as a substitute for natural coarse aggregate in cement composites in terms of environmental safety, mechanical properties, the solidification effect of the cement matrix, and the life-cycle assessment. Based on the results obtained, the following conclusions can be drawn:

1. The environmental characterization of recycled brick material demonstrated significant variability in the properties of recycled materials originating from different recycling yards. Higher concentrations of arsenic, chromium, EOX, and PAHs were detected in the dry matter of selected samples, while elevated concentrations of dissolved substances and sulfates were recorded in the leachates. The results confirm the need for environmental assessment of recycled materials prior to their further use. For engineering applications, the incoming quality control of recycled brick aggregate should therefore include, as key indicators, material composition and particle-size distribution, water absorption, particle density, and, for the coarse fraction, the shape index. Environmental quality control should include the assessment of the leachate, particularly dissolved solids, sulfates, chlorides, DOC, and potentially relevant heavy metals, as well as dry-matter analysis of relevant contaminants, particularly As, Cr, EOX, and PAHs. The specific scope of testing should be selected according to the intended application and applicable environmental requirements.
2. The fresh properties of cement composites were influenced by the extent to which natural aggregate was replaced with recycled brick material. As the proportion of recycled material increased, the workability and bulk density of the fresh concrete decreased. Nevertheless, all designed mixtures had the properties suitable for practical application.
3. Under the material and mixture-design conditions investigated in this study, complete replacement of natural aggregate by RBA was feasible while maintaining a 28-day compressive strength of 34.7 MPa. This finding demonstrates the potential for high RBA replacement levels; however, its applicability to other concrete mixtures depends on the properties of the recycled aggregate and the specific mixture design.
4. A significant solidification effect of the cement matrix was demonstrated. Compared to the original recycled brick material, concentrations of dissolved substances were reduced by 68.0–80.1% and sulfate concentrations by 97.8–99.2%. All proposed cement composites also met the limit values specified in Decree No. 273/2021 Coll.
5. The cement matrix effectively immobilized the potentially mobile components contained in the recycled brick material. The concentrations of most of the monitored elements and compounds in the leachates from the hardened cement composites were below the limit of quantification of the analytical methods used or significantly below the legislative limits.
6. The life cycle assessment demonstrated a gradual reduction in environmental impacts as the proportion of recycled brick material increased. The mixture containing 100% recycled brick material achieved the lowest values in most of the evaluated environmental categories, including global climate change potential and fossil resource consumption.
7. Image analysis demonstrated good compatibility between the recycled brick aggregate and the cement matrix. In all evaluated cement composites where natural coarse aggregate was replaced by recycled brick aggregate in the range of 50–100%, grains of natural aggregate, grains of recycled brick aggregate, and cement paste were clearly identified. At the same time, no disruption of the interfacial transition zone (ITZ) between the aggregate grains and the cement paste was observed, nor were any cracks found in the cement matrix. The results of the image analysis confirm a high-quality bond between the recycled aggregate and the cement matrix, which corresponds to the mechanical properties achieved by the experimental mixtures.
8. The results confirm that recycled brick material can be safely used as a full-fledged substitute for natural coarse aggregate in cement composites. This approach contributes to the conservation of primary mineral resources, supports the principles of the circular economy in the construction industry, and enables the production of environmentally acceptable cement composites with satisfactory mechanical properties.

## Figures and Tables

**Table 1:** Physical properties of recycled brick aggregate RD3 (0/22.4 mm).

Parameter	Unit	Value
Fraction of recycled material	mm	0/22.4
Bulk density o grains	kg·m^−3^	2120
Water absorption WA24	%	10.6
Percentage of particles < 0.25 mm	%	5
Percentage of particles < 4 mm	%	35
Percentage of particles < 8 mm	%	53
Percentage of particles < 16 mm	%	87
Maximum grain size D	mm	22.4
Shape index (4/8 mm)	%	12.1
Shape index (8/16 mm)	%	12.5
Shape index (16/22 mm)	%	12.5
Shape index category	–	SI15

**Table 2:** Basic properties of Žabčice natural aggregate fraction 0/4 mm.

Parameter	Unit	Value
Aggregate fraction	Mm	0/4
Grain size category	–	GF85
Bulk density of grains	Mg·m^−3^	2.56
Water absorption WA24	%	1.19
Fine particle content	–	f3
Chloride content	%	0.0001
Sulfate content	–	AS0.2
Total sulfur content	–	compliant (<1%)
Humic substance content	–	compliant
Natural radioactivity	–	Ra226 < 15

**Table 3:** Selected properties of CEM I 42.5 R cement.

Parameter	Unit	Value
Cement type	–	CEM I 42.5 R
Portland clinker content	%	95–100
Content of additives	%	0–5
Specific gravity	kg·m^−3^	3130
Specific surface area (Blaine)	m^2^·kg^−1^	303
Normal consistency	%	28.0
Initial setting	Min	228
Final setting	Min	320
Volume stability	Mm	0.7
Compressive str. after 2 days	MPa	27.1
Compressive str. after 28 days	MPa	59.2
Hydration heat (7 days)	J·g^−1^	319
Content of SO_3_	%	3.09
Content o Cl^−^	%	0.027
Na_2_O equivalent	%	0.63

**Table 4:** Composition of the reference and experimental cement composite mixtures.

Component [kg/m^3^]	Replacement of Natural Aggregate with Recycled Rick Material [%]				
	REF 0	50	70	80	100
CEM I 42.5 R	315	305	305	305	305
Natural aggregate 0/4	981	846	518	356	-
Natural aggregate 8/16	870	-	-	-	-
Recycled brick material 0/22	-	709	1016	1192	1451
Water	176	265	246	243	270
Plasticizing additive Quad 920	0.8	4.5	4.5	4.5	4.5
Water-to-cement ratio (w/c)	0.56	0.87	0.81	0.80	0.89

**Table 5:** Concentrations of selected indicators in the dry matter of recycled brick materials collected from recycling yards RD1–RD4.

Indicator	I. Limit Value [mg·kg^−1^ dry mat.]	II. Limit Value [mg·kg^−1^ dry mat.]	RD1 0/4	RD2 0/4	RD3 0/4	RD4 0/4	RD1 4/22.4	RD2 4/22.4	RD3 4/22.4	RD4 4/22.4
As	10	30	11	11	12	16	12	9	12	12
Cr	100	200	477	282	231	230	333	275	191	184
EOX	1	2	5	0	5	0	3	0	6	0
PAU	3	6	0	0	0	9	0	0	0	2

**Table 6:** Concentrations of selected indicators in aqueous extracts of recycled brick materials.

Indicator	Limit Value [mg·L^−1^]	RD1 0/4	RD2 0/4	RD3 0/4	RD4 0/4	RD1 4/22.4	RD2 4/22.4	RD3 4/22.4	RD4 4/22.4
RL	400	819	621	827	652	387	351	536	368
Sulfates	100	381	272	393	358	156	122	277	238
Fluorides	1.0	0.70	0.00	0.00	0.76	1.07	0.00	0.00	0.47

**Table 7:** Physical and mechanical properties of the reference and experimental cement composite mixtures.

		Replacing Natural Aggregate with Recycled Brick Aggregate [%]				
		REF 0	50	70	80	100
Determination of the consistency of fresh concrete	[mm]	240	150	170	150	110
Determination of the air content in fresh concrete	[%]	2.1	2.8	3.0	3.2	3.3
Determination of the bulk density of fresh concrete	[kg/m^3^]	2345	2130	2090	2100	2030
Determination of the bulk density of hardened concrete	[kg/m^3^]	2310	2040	1990	2020	1950
Compressive strength after 28 days	[MPa]	34.5	36.2	31.8	33.6	34.7

Note: The reported 28-day compressive strength values represent the arithmetic mean of three test specimens (*n* = 3) for each mixture.

**Table 8:** Comparison of the present study with selected previous investigations on concrete containing recycled brick aggregate.

Study	RBA Replacement Level	RBA Type/Investigated Parameter	w/c	Slump/ Workability	28-Day Compressive Strength	Effect of RBA on Concrete Properties
Debieb and Kenai [1]	25, 50, 75, 100%	Fine, coarse, or combined crushed brick aggregate	Varied to maintain the required workability	60–70 mm	Generally decreased with increasing replacement level	Comparable properties were obtained at limited replacement levels; mixture behaviour was strongly affected by aggregate conditioning and water demand
Cachim [2]	15, 30%	Crushed brick aggregate; two brick types	0.45; 0.50	≈50 mm at w/c = 0.45; ≈150 mm at w/c = 0.50	No strength reduction at 15% replacement; reduction of up to ≈20% at 30%, depending on brick type	Workability was maintained approximately constant for each w/c by accounting for aggregate water absorption
González et al. [3]	20, 35, 50, 70, 100%	Fine and coarse recycled ceramic brick aggregate	Not reported in the source used for comparison	Not reported in the source used for comparison	Progressive reduction with increasing replacement; approximately 23% reduction at 100% replacement compared with reference concrete	Structural concrete was feasible; for the investigated prestressed concrete application, replacement levels up to 35% were recommended
Uddin et al. [4]	Conventional replacement series not investigated	Coarse brick aggregate; maximum aggregate size 12.5–50 mm	0.45; 0.50; 0.55	Dependent on w/c, cement content, sand-to-aggregate ratio, and maximum aggregate size	Non-monotonic response depending on maximum aggregate size and mixture parameters	Compressive strength was governed by a combination of mixture-design parameters and aggregate characteristics rather than by a single aggregate parameter
Present study	50, 70, 80, 100%	RBA 0/22.4 mm; progressive replacement of natural aggregate	0.87; 0.81; 0.80; 0.89	150; 170; 150; 110 mm	36.2; 31.8; 33.6; 34.7 MPa	All RBA mixtures exceeded 30 MPa; the mixture with 100% RBA reached 34.7 MPa, demonstrating a non-monotonic relationship between RBA replacement level and compressive strength

**Table 9:** Comparison of selected parameters in the leachate of RD3 recycled brick material (0/22.4 mm fraction) and cement composites with varying degrees of natural aggregate replacement.

Indicator	Limit Value [mg·L^−1^]	Recycled Bricks RD3 fr. 0/22.4 mm	Recycled Bricks (50%)	Recycled Bricks (70%)	Recycled Bricks (80%)	Recycled Bricks (100%)
RL	400	638	204	163	127	135
Sulfates	100	318	5.85	3.80	2.57	6.88
Fluorides	1.0	<0.02	<0.02	<0.02	<0.02	<0.02
Cr total	0.05	0.044	<0.030	<0.030	<0.030	<0.030
RL solidification effic. [%]	–	–	68.0	74.4	80.1	78.8
Sulfate solidification efficiency [%]	–	–	98.2	98.8	99.2	97.8

Note: The values for RD3 recycled aggregate in the 0/22.4 mm fraction were determined as the weighted average of the concentrations found in the 0/4 mm and 4/22.4 mm fractions, using the proportions of the individual fractions determined by grain size distribution analysis (35% for the 0/4 mm fraction and 65% for the 4/22.4 mm fraction).

**Table 10:** Environmental impacts of the investigated concrete mixtures in the product stage (A1–A3) and contributions of the subsequent life-cycle modules.

Environmental Impact Category	Unit	0%	50%	70%	80%	100%	A4	A5	B	C1	C2	C3	C4	D
Climate change— Total	kg CO_2_ eq	303	299	296	295	293	9.34	3.35 × 10^−2^	−10.0	2.84	11.2	9.24	0.658	−38.8
Ozone depletion	kg CFC-11 eq	6.29 × 10^−6^	5.10 × 10^−6^	4.53 × 10^−6^	4.24 × 10^−6^	3.67 × 10^−6^	2.03 × 10^−7^	2.54 × 10^−10^	0	4.17 × 10^−8^	2.44 × 10^−7^	1.37 × 10^−7^	1.83 × 10^−8^	−4.09 × 10^−7^
Acidification	mol H^+^ eq	0.598	0.587	0.580	0.576	0.569	2.00 × 10^−2^	1.55 × 10^−4^	0	9.79 × 10^−3^	2.40 × 10^−2^	8.25 × 10^−2^	4.60 × 10^−3^	−0.243
Eutrophication, freshwater	kg P eq	3.84 × 10^−2^	3.66 × 10^−2^	3.57 × 10^−2^	3.53 × 10^−2^	3.44 × 10^−2^	6.47 × 10^−4^	4.92 × 10^−5^	0	9.04 × 10^−5^	7.77 × 10^−4^	2.97 × 10^−4^	5.75 × 10^−5^	−9.68 × 10^−3^
Resource use, fossils	MJ	1430	1360	1330	1310	1270	133	0.546	0	36.6	159	120	16.1	−509
Water use	m^3^ depriv.	27.5	26.4	25.9	25.6	25.1	0.679	1.04 × 10^−2^	0	0.106	0.815	0.348	0.714	−33.0

Note: The values for 0%, 50%, 70%, 80%, and 100% recycled brick aggregate replacement represent the product stage (A1–A3) for the respective concrete mixtures. The values for the subsequent life-cycle modules (A4, A5, B, C1–C4, and D) are identical for all investigated mixtures because the same scenarios for transport, installation, use stage, and end-of-life were assumed. Negative values indicate environmental benefits or credits.

## Data Availability

The original contributions presented in this study are included in the article. Further inquiries can be directed to the corresponding author.
